# Immunomodulatory properties of the lymphatic endothelium in the tumor microenvironment

**DOI:** 10.3389/fimmu.2023.1235812

**Published:** 2023-09-07

**Authors:** Cristina Viúdez-Pareja, Ewa Kreft, Melissa García-Caballero

**Affiliations:** ^1^Department of Molecular Biology and Biochemistry, Faculty of Sciences, Andalucía Tech, University of Málaga, Málaga, Spain; ^2^IBIMA (Biomedical Research Institute of Málaga)-Plataforma BIONAND, Málaga, Spain

**Keywords:** tumor microenvironment, lymphatic vasculature, lymphatic endothelial cells, immune system, tumor-draining lymph node, pre-metastatic niche, metastasis

## Abstract

The tumor microenvironment (TME) is an intricate complex and dynamic structure composed of various cell types, including tumor, stromal and immune cells. Within this complex network, lymphatic endothelial cells (LECs) play a crucial role in regulating immune responses and influencing tumor progression and metastatic dissemination to lymph node and distant organs. Interestingly, LECs possess unique immunomodulatory properties that can either promote or inhibit anti-tumor immune responses. In fact, tumor-associated lymphangiogenesis can facilitate tumor cell dissemination and metastasis supporting immunoevasion, but also, different molecular mechanisms involved in LEC-mediated anti-tumor immunity have been already described. In this context, the crosstalk between cancer cells, LECs and immune cells and how this communication can shape the immune landscape in the TME is gaining increased interest in recent years. In this review, we present a comprehensive and updated report about the immunomodulatory properties of the lymphatic endothelium within the TME, with special focus on primary tumors and tumor-draining lymph nodes. Furthermore, we outline emerging research investigating the potential therapeutic strategies targeting the lymphatic endothelium to enhance anti-tumor immune responses. Understanding the intricate mechanisms involved in LEC-mediated immune modulation in the TME opens up new possibilities for the development of innovative approaches to fight cancer.

## 1 Introduction

Tumor growth and progression are driven by complex interactions between cancer cells and their microenvironment. The tumor microenvironment (TME) is defined as a dynamic network of cancer cells, non-cancerous cells, extracellular matrix components, and signaling molecules that interact with tumor cells, shaping their behavior and consequently, influencing their response to therapies (1). It is increasingly recognized that the TME plays a crucial role in cancer development, progression, and metastasis, which opens new field of research targeting TME as a therapeutic strategy (1). A key player of the TME are lymphatic vessels (LVs).

In physiological conditions, LVs participate in the maintenance of the tissue fluid homeostasis, immune cell trafficking and the absorption of dietary lipids in the intestine (2). However, dysfunction of the lymphatic system or deregulation of the lymphangiogenic process (formation of new LVs from preexisting ones) have been associated with multiple diseases (3, 4). For instance, impaired lymphatic function is linked to lymphedema, a disorder characterized by extensive swelling due to improper drainage of fluid that accumulates in tissues of limbs (5). In the context of inflammatory processes, the lymphatic vascular network appears to be expanded due to lymphatic hyperplasia and abnormal lymphangiogenesis (6), as shown in both mouse models and patient samples of inflammatory diseases, such as arthritis (7), atopic dermatitis (8), psoriasis (9), and inflammatory bowel disease (10). Distorted function of LVs has been also associated with cardiometabolic diseases (11), infection diseases (12), and more recently, neurodegenerative pathologies (13).

In the context of the TME, tumor-associated LVs can play a dual role (14). On one hand, LVs provide cancer cells with a way of dissemination to lymph nodes (LNs), leading to the formation of pre-metastatic and metastatic niches (15, 16), and finally, the development of lymphatic metastasis, which is linked to poor patient prognosis (17, 18). On the other hand, it has been recently described that LVs can be responsible for the improvement of immune response against the tumor and allow more efficient delivery of chemotherapy (19).

Considering the role of the immune system in tumor progression, it is worth highlighting the ability of tumor cells to evade the host’s immune system, allowing for unchecked growth and survival, which is known as the escape of the immune surveillance, and constitutes one of the hallmarks of cancer (20). However, not only cancer cells can participate in the immune trafficking. Within the TME, lymphatic endothelial cells (LECs), which are specialized endothelial cells (ECs) lining LVs, are an integral part and have been shown to play a role in the regulation of immune cell trafficking and the development of immunosuppressive pathways that can promote tumor progression (14). More recently, it has been described that their unique molecular identity accounts for the ability to interact with tumor cells and other elements of the TME, having dual effects, as they might both promote and suppress tumor growth (21). As such, understanding the mechanisms underlying cancer cell-LEC-immune cell crosstalk, as well as the immune modulating functions of LECs, may offer new perspectives in cancer research, providing opportunities for the development of novel therapies targeting the TME and promoting the induction of anti-tumor immune responses.

In this review, we highlight the role of LECs in tumor development and progression, and describe the immunomodulatory properties of the lymphatic endothelium in the complex interplay between tumor cells and the immune system. We also present recent advances that reveal the potential of targeting the lymphatic endothelium as a therapeutic strategy against cancer.

## 2 Lymphatic system

The lymphatic system is a network of vessels and tissues primarily responsible for maintaining fluid balance and regulation of the body’s immune response (2). The components that account for these functions include a network of LVs and secondary lymphoid organs, such as LNs or mucosa-associated lymphatic tissue (MALT), that spread throughout the body (2).

**Figure 1:**
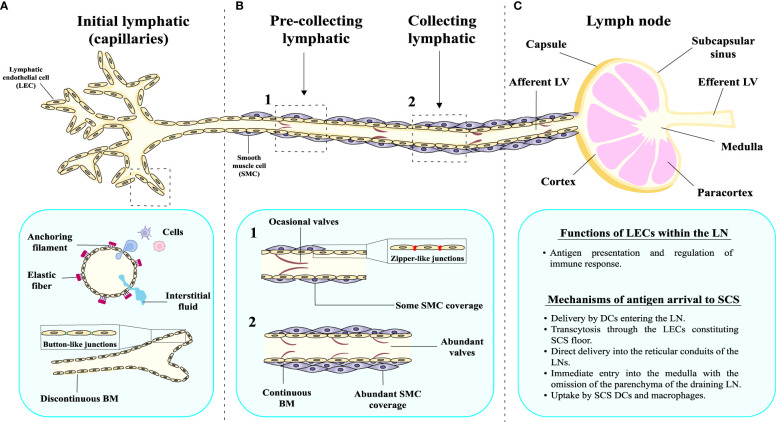
Morphological and structural characteristics of the lymphatic vessels and LNs. The lymphatic system comprises an intricate network of lymph nodes interconnected by lymphatic vasculature, exhibiting distinct structural characteristics at various levels of organization. **(A)** Initial lymphatics or capillaries present a discontinuous basement membrane (BM), which facilitates the permeation of fluid into the lumen. Button-like junctions can be observed, serving as critical points of connection between adjacent cells. They also have anchoring filaments on their outer surface. **(B)** Pre-collecting lymphatic vessels have a different structure, since the BM appears relatively continuous, providing a more consistent barrier. Moreover, the pre-collecting LVs are surrounded by smooth muscle (SM) cells, contributing to their contractile properties, and the emergence of valves within the pre-collecting lymphatics becomes evident. In case of collecting LVs, the BM is completely continuous, multiple valves are present and the vessel is surrounded by thick layer of SM cells. **(C)** Collecting LVs drain to a lymph node, which has different anatomical parts and is involved in important physiological functions.

The morphology of LVs differs among vessels of different magnitudes that also perform distinct functions (**Figure 1**). Initial lymphatics or capillaries are comprised of blind-ended sacs with a scarce basal membrane and a single layer of LECs with thin and discontinuous walls. The walls of lymphatic capillaries exhibit button-like junctions that help the interstitial fluid and cells enter the lumen of the vessel (**Figure 1A**). Moreover, initial LVs possess anchoring filaments on their outer surface that are responsible for connecting the vessels to the elastic fibers present in the surrounding tissues (**Figure 1A**). On the other hand, pre-collecting LVs are conduits arising from initial lymphatics that connect these capillaries to the collecting vessels (**Figure 1B**). They are characterized by the presence of occasional valves, fenestrated basal membrane, zipper-like junctions and scarce smooth muscle cell coverage (**Figure 1B**). In contrast, collecting lymphatics are vessels with complete basal membrane, fully covered by a layer of smooth muscle cells and with often recurring valves that serve a purpose of preventing back flow of the lymph (2) (**Figure 1B**).

The interior surface of LVs (capillaries, pre-collecting and collecting vessels) is lined by a single layer of LECs that constitutes the lymphatic endothelium. During development, blood endothelial cells (BECs) are differentiated into arterial and venous EC subsets, in a process controlled by Notch signaling pathway (22). Venous ECs further undergo the differentiation into LECs in a process strictly controlled by the expression of the *Prospero homeobox protein 1* (*PROX1*), *SRY-Box Transcription Factor 18 (SOX18)* and *NR2F2* (22). Although LECs share common properties with BECs, they have distinct gene expression profiles that reflect their different functions and tissue specificity (23, 24). For instance, BECs express genes involved in the formation and maintenance of blood vessels, such as *Endoglin*, the *Vascular Endothelial Growth Factor Receptor (VEGFR)-1* and *Angiopoietin* 1 (*ANGPT1*), as well as proteins involved in signaling pathways that regulate blood flow and vascular permeability, such as NADPH Oxidases (NOX) and Rho GTPases (25, 26), among others (**Table 1**). In contrast, LECs express genes involved in the formation and maintenance of LVs, such as *PROX1*, *Podoplanin (PDPN)*, *Lymphatic vessel endothelial hyaluronan receptor 1* (*LYVE1*) and *FLT4* (2) (**Table 1**). Some marker genes are found in both blood and LVs, such as *Platelet and Endothelial Cell Adhesion Molecule 1 (PECAM1)* and *CD34* (44) (**Table 1**). Of note, a more complete list with the common specific and markers between LECs and BECs is displayed in **Table 1** (27–56).

**Table 1:** Distinct and shared marker genes between lymphatic endothelial cells (LECs) and blood endothelial cells (BECs), and their corresponding functions.

Cell type	Marker	Function	Reference
BECs	ANGPT1	Regulation of angiogenesis	(27)
	NOX	Host defense, post-translational processing of proteins, cellular signaling regulation of gene expression, and cell differentiation	(25, 28)
	Rho GTPases	Control of actin cytoskeleton	(26, 29)
	CD44	Control of vascular integrity, proliferation, and apoptosis of endothelial cells	(30)
	VEGFR-1/FLT1	VEGF-A/B and PLGF receptor involved in the control of angiogenesis	(31)
	Endoglin/CD105	TGF-β co-receptor involved in proliferation, control of apoptosis and regulation of angiogenesis	(32)
	IL-8	Chemoattractant of endothelial cells and neutrophils during angiogenesis	(33)
LECs	PROX1	Promotion of lymphangiogenesis, determination of the lymphatic fate by the initiation of the lymphatic differentiation program	(2, 34)
	PDPN	Control of development of the lymphatic system, regulation of cell motility	(2, 35)
	LYVE1	Receptor of hyaluronic acid, control of cell trafficking and migration	(2, 36)
	VEGFR-3/FLT4	VEGF-C/D receptor, control of lymphangiogenesis and LEC proliferation	(2, 37)
	CCL21/SCL	Guiding of CCR7-expressing cells into lymphatic vessels	(38)
	Desmoplakin	Component of desmosomes involved in the interaction with intermediate filaments	(39)
	Integrin α9	Mediation of migration, lymphangiogenesis and lymphatic valve morphogenesis	(40)
	MRC1	Lymphocyte trafficking	(41)
	Dipeptidyl peptidase IV	Membrane glycoprotein involved in cell differentiation, apoptosis, and binding to collagen, fibronectin and gelatin	(42)
BECs & LECs	PECAM1/CD31	Control of vascular permeability and integrity of endothelial cell junctions	(43, 44)
	CD34	Development of vessels, promotion of lymphocyte adhesion	(44, 45)
	NRP1 and NRP2	VEGFRs co-receptors involved in angiogenesis and lymphangiogenesis	(42, 46)
	VEGFR2	VEGF-A/C/D receptor involved in the control of vasculogenesis, angiogenesis and lymphangiogenesis	(31)
	CCL20	Recruitment of CCR6+ cells	(47)
	VE-Cadherin	Maintenance of junctional vessel integrity and control of permeability	(48, 49)
	JAM-2	Ligand for immune cells, possible role in lymphocyte homing in secondary lymphoid structures	(50, 51)
	E-Selectin	Leukocyte rolling mediator	(52)
	ICAM-1	Regulation of leukocyte trafficking	(53)
	CD146	Adhesion molecule involved in angiogenesis, lymphangiogenesis and vessel integrity	(54)
	Collagen IV	Component of basal membrane, control of adhesion and migration, regulation of angiogenesis	(55)
	Collagen XVIII	Component of basal membrane, maintenance of basal membrane integrity, regulation of cell survival and differentiation	(56)

The features responsible for the distinct properties of LECs include fenestrations, contractile properties, which propels the fluid through the LVs, and the capability to absorb excess of fluid from the interstitial spaces and transport it back to the bloodstream. Moreover, the direct contact with the lymph, a fluid with a composition different to that of the blood, determines the different characteristics of both EC types.

Another important player in the lymphatic system are the LNs (57) (**Figure 1C**). Mammalian LNs can be characterized by several afferent LVs, a complex system of intranodal vasculature and an efferent LV (58). The components of a typical human LN include follicles and interfollicular cortex surrounded by the mesh of paracortical sinuses (58). From a structural point of view, the parts of the human LN are: capsule, subcapsular sinus, cortex, paracortex and medulla (**Figure 1C**) (59). This structure is crucial for performing their function, as they allow the entrance of antigens by different mechanisms. The best-known mechanism of antigen presentation is based on dendritic cells (DCs) entering the LN and delivering the antigen to T lymphocytes within the LN parenchyma (4, 58, 60). However, in certain cases, antigens with specific chemical characteristics present in the lymph, known as free antigens, are able to directly enter the LN (61). In fact, studies in mice have demonstrated the existence of different mechanisms through which a subcutaneously injected antigen can arrive at the subcapsular sinus (SCS) of a LN. These mechanisms include: (1) migration (transcytosis) through the LECs constituting the SCS floor (62); (2) direct delivery into the reticular conduits of the LNs (63, 64); (3) immediate entry into the medulla with the omission of the parenchyma of the draining LN (65, 66); and (4) uptake by SCS DCs and macrophages (67). As LECs constitute integral part of the SCS, they are directly involved in the management of the antigen input into the LN, playing a key role in the antigen presentation process to immune cells and the regulation of immune responses (**Figure 1C**).

## 3 LEC immune profiling

**Figure 2:**
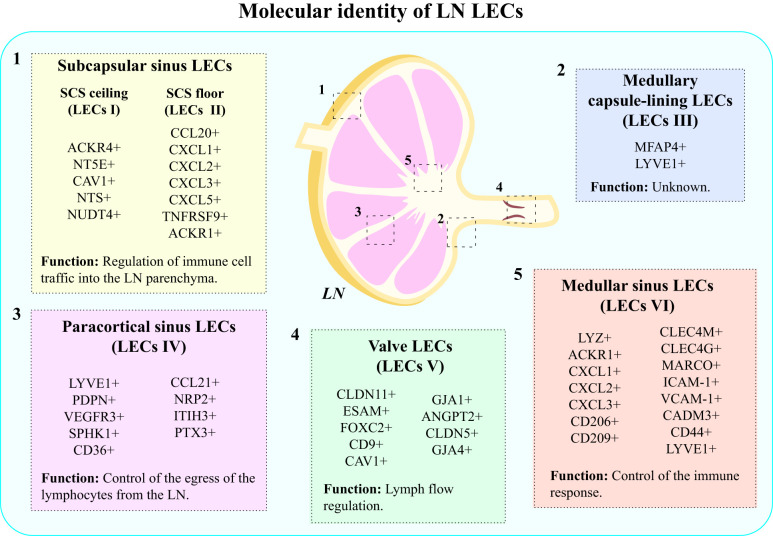
Molecular identity of LN LECs. LECs present in LNs display heterogeneity, which results from different molecular markers expressed by distinct subpopulations of LECs. The main types of LECs present in human LNs include: (1) subcapsular sinus LECs: SCS ceiling LECs (LECs I) and SCS floor LECs (LECs II); (2) medullary capsule-lining LECs (LECs III); (3) paracortical sinus LECs (LECs IV); (4) valve LECs (LECs V); and (5) medullary sinus LECs (LECs VI).

LECs comprise the basic structural components of the LN sinus lining, and the vasculature they form within the LNs displays distinct phenotype and function in comparison to afferent LVs and peripheral lymphatic capillaries (58, 68). In humans, LECs present in the LNs can be categorized into 6 subsets: (1) SCS ceiling LECs (LECs I in human, cLECs in mouse), (2) SCS floor LECs (LECs II in human, fLECs in mouse), (3) medullary capsule-lining LECs (LECs III in human), (4) paracortical sinus LECs (LECs IV in human, Ptx3-LECs in mouse), (5) Valve LECs (LECs V in human) and (6) medullary sinus LECs (LECs VI in human, Marco-LECs in mouse) (58, 68) (**Figure 2**).

There are significant differences between molecular markers expressed by cells belonging to different subsets, which account for the different functions they perform (58, 68) (**Figure 2**). For instance, LECs I express atypical chemokine receptor 4 (ACKR4), which serves a purpose of creating gradient for CCR7 ligands, and thus regulating DC migration into LN parenchyma (68). Besides that, they participate in the absorption of acetylated low-density lipoprotein (LDL) molecules (69) and synthesis of CD73 (5’-nucleotidase ecto, NT5E) –an enzyme converting AMP to adenosine, which possesses anti-inflammatory properties (70). LECs I also express caveolin 1 (CAV1), NTS and NUDT4 (71). On the other hand, LECs II express a wide range of inflammatory chemokines, such as CCL20, CXCL1, CXCL2, CXCL3, and CXCL5, TNFRSF9, and ACKR1 (58, 71). Their primary function is the regulation of immune cell traffic into the LN parenchyma (68, 69). LECs III are characterized by the expression of microfibril associated protein 4 (MFAP4) and LYVE1. Interestingly, LECs III, found in the capsule lining of the medulla do not have the murine equivalent, and their exact function remains unknown (58). LECs IV exhibit high expression of LYVE1, PDPN, VEGFR-3, sphingosine kinase 1 (SPHK1), CD36, neuropilin 2 (NRP2), inter-alpha-trypsin inhibitor (ITIH3) and CCL21 (68). LECs of this subset participate in the control of the egress of lymphocytes from the LN (68, 69). Besides that, they selectively express pentraxin 3 (PTX3), which triggers the classical pathway of the complement and induces immune response (72). LECs V form lymphatic valves, which are essential for lymph flow (69), and are characterized by the expression of CLDN11, ESAM and Forkhead box protein C2 (FOXC2) (58, 68, 71). LECs composing upstream and downstream sides of valves have been shown to express different markers: LECs in the upstream side express CD9, CAV1 and gap junctional protein α1 (GJA1 or connexin-43), while in the downstream side, LECs express ANGPT2, CLDN5 and GJA4 (or connexin-37) (71, 73). These cells seem to derive from collecting lymphatics (71). Medullary sinus LECs (LECs VI) share the expression of several genes with SCS floor LECs (LECs II), and they are characterized by the expression of lysozyme (LYZ), ACKR1, neutrophil chemoattractants (CXCL1, CXCL2, CXCL3), C-type lectins (CD206, CD209, CLEC4M, CLEC4G), macrophage receptor with collagenous structure (MARCO), adhesion molecules (ICAM-1, VCAM-1, CADM3 and CD44) and LYVE 1 (58, 71). The expression of neutrophil chemoattractants and, particularly, of CD209, shows that these cells have a role in neutrophil migration and recruitment to LNs (71). Expression of MARCO has been suggested to be involved in the control of virus dissemination (74, 75). Therefore, the molecular signature of LECs VI indicates the involvement of these cells in the control of the immune response (**Figure 2**).

The important immunomodulatory properties of LECs are executed through complex cellular interactions mediated by chemokines. LECs express both chemokine ligands and receptors that influence their behavior in both physiological and pathological lymphangiogenesis, and determine the immune cell trafficking (76). For instance, CCL2, a ligand targeting the CCR2 receptor present on the surface of macrophages, plays a substantial role in developmental lymphangiogenesis (77). CCL21, a ligand for CCR7, is involved in the crosstalk between LECs and activated DCs, neutrophils and T lymphocytes (78–80). As such, it regulates the migration of these cells into initial lymphatics and guides them through the lymphatic vasculature to LNs and within LNs. Other ligands playing roles in controlling trafficking of immune cells into the LNs include CCL20, CXCL12 and CX3CL1 (81–83). Moreover, specific subsets of LECs (cLECs, fLECs and Marco-LECs) have been shown to be involved in the storage of antigens in mice (84). This allows for the controlled release of antigens within the LN, and the antigen presentation to DCs after the antigen has already reached the LN. This mechanism is potentially useful in the long-term perspective of controlling pathogen-induced infections.

McKimmie et al. found that ACKR2, also known as D6, selectively found on LECs, is regulated by growth factors and cytokines in the TME, and plays a role in the recognition of mature DCs by LECs (85). Overexpression of ACKR2 reduces the adhesion of immature DCs (iDCs) to LECs, while reducing D6 levels increases the adhesion of iDCs and displaces mature DCs (85). Moreover, scavenging receptor D6 plays a role in this process by suppressing the binding of inflammatory chemokines to LEC surfaces. This helps prevent inappropriate attachment of inflammatory cells to LECs (85).

Overall, distinct LEC subtypes are distributed across diverse locations within the lymphatic system. These subpopulations of LECs display distinct immunomodulatory and molecular identities, which determine their properties and functional attributes.

## 4 Immunomodulatory properties of LECs in the tumor microenvironment

As mentioned above, LECs produce and respond to various types of cytokines, playing a significant role in modulating immune responses through facilitation of antigen presentation and immune cell activation (76, 86). It is worth noticing that one of the factors influencing the complexity of these interactions is the fact that, in certain cases, LECs express both ligands and their receptors (76). For instance, CXCL12 expressed on the LEC surface can target the same CXCR4 receptor present on different cell types. If CXCL12 binds to CXCR4 expressed by DCs, the immune trafficking processes are triggered, but if it binds to CXCR4 present on cancer cells, the LN metastasis cascade begins (87). Nevertheless, when LECs express CXCR4 that later interacts with CXCL12 found on tumoral cells, tumor lymphangiogenesis and lymphatic metastasis are promoted (87). Recently, it has been found that G Protein-Coupled Receptor 182 (GPR182), which is expressed in LECs, might belong to the ACKR family and plays a role in controlling the presence of chemokines CXCL9, CXCL10 and CXCL11 in the TME, therefore controlling the immune response (88).

But the immune profile is not exclusive of LECs, since other EC types like BECs also express immune-related genes, such as chemokines, cytokines, Co-stimulatory adhesion molecules, which are different among tissues and organs, resulting in tissue-specific immunomodulation (89). For instance, blood vessels provide a route for the arrival of immune cells to the tumor, and through the expression of adhesion molecules (selectins and integrins), and the secretion of chemokines and cytokines, BECs participate in immune cell activation, migration, and recruitment, and play a crucial role during the extravasation of immune cells (89). When activated, BECs upregulate the expression of adhesion molecules and produce cytokines such as IL-6, IL-8, CCL21, SDF, and macrophage inflammatory protein (MIP3) alpha and beta (90, 91), all of which contribute to leukocyte trafficking. Interestingly, immune cell extravasation in both blood and lymphatic vessels occurs in a similar way and involves the expression of VCAM-1, ICAM-1, and E-selectins (91, 92). However, tumor-associated blood vessels are dysfunctional and leaky (93), and the continuous exposure of BECs to pro-angiogenic factors can lead to endothelial anergy, a state in which ECs stop responding to inflammatory signals (90). This affects leukocyte trafficking negatively, giving rise to an immunosuppressed microenvironment (91).

Another immunomodulatory property shared by LECs and BECs is their ability to process and present antigens to immune cells (89). BECs and LECs actively capture antigens from the surrounding microenvironment, and following intracellular processing, these antigens are presented on their surfaces via MHC-I and MHC-II molecules (94). On the other hand, BECs and LECs, do not normally express the co-stimulatory molecules CD80 and CD86, therefore acting as semi-professional antigen presenting cells (89). In addition, BECs and LECs express programmed cell death protein 1 ligand (PD-L1) and can modulate T cell activation through the PD-L1/PD-1 pathway (90, 91).

In the context of cancer and the TME, LECs, and also BECs, can play a dual role, acting both as facilitators and suppressors of the immune response (21), and displaying a dynamic role on antigen presentation, immune cell trafficking and the modulation of the immune response. One of the key immunological functions of LECs in the TME is the regulation of LV formation, enabling LN metastasis and formation of pre-metastatic niches (16). Another aspect of the tumor promoting LEC activity is their ability to produce immunosuppressive cytokines, such as transforming growth factor (TGF)-β, that can weaken the immune response (95). LECs can also produce immune checkpoint inhibitors, such as PD-1, that can inhibit T cell activation and function (96). All in all, by facilitation or evasion of immune cell trafficking to the site of the tumor, LECs can both promote and suppress anti-tumor immunity. In the following subsections, the mechanisms underlying the dual role of LECs in tumor immunity are described in detail.

### 4.1 Pro-tumor immune mechanisms of LECs

**Figure 3:**
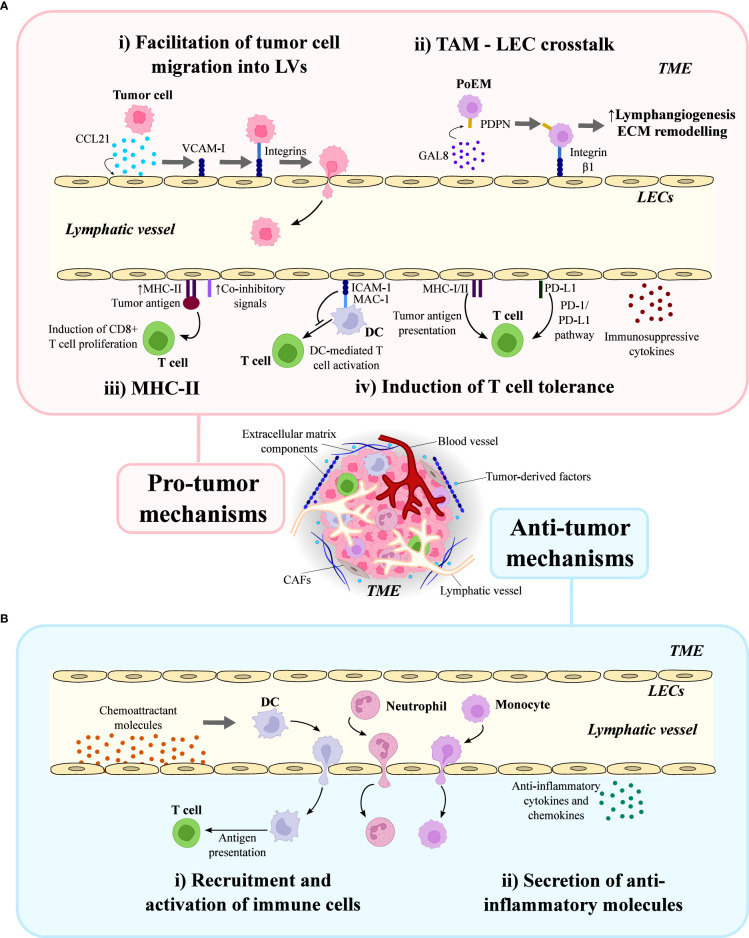
Dual role of lymphatic endothelial cells (LECs) in the tumor microenvironment (TME). LECs in the TME can act as facilitators and suppressors of the immune response, playing a dual role in tumor immunity. **(A)** LECs are involved in pro-tumor mechanisms: (i) they facilitate tumor adhesion and migration into lymphatic vessels, a process in which CCL21, VCAM-I and integrins are involved; (ii) they establish a crosstalk with tumor-associated macrophages (TAMs) through galectin 8 (GAL8), podoplanin (PDPN) and integrin β1, which promote lymphangiogenesis and extracellular matrix (ECM) remodeling in the TME; (iii) they promote immunosuppression in a MHC-II-dependent manner, increasing the co-inhibitory signals and inducing CD8^+^ T cell proliferation; and (iv) they are involved in the T cell tolerance induction through DC-mediated T cell activation and release of immunosuppressive cytokines. **(B)** LECs are also involved in anti-tumor mechanisms: (i) they recruit dendritic cells (DC), neutrophils and monocytes by producing chemoattractant molecules; and (ii) they secrete anti-inflammatory cytokines and chemokines in the TME.

Basic mechanism through which LECs can directly promote tumor cell migration and invasion is by providing a route for cancer cells to enter the LVs and disseminate to distant sites (21). While these processes arise from specific properties of LECs, they are conditioned by multiple interactions between them and tumor cells in their proximity. Certain tumor-derived chemokines such as CCL21 can activate LECs and increase the expression of adhesion molecules (78, 97) (**Figure 3A**). Presence of ICAM-1, VCAM-1 and E-selectins on LECs enables the interaction with integrins present on tumor cells, facilitating their adhesion and migration through the lymphatic endothelium, and thus enabling their entrance into LVs (92, 98) (**Figure 3A**). It has also been shown how tumor cells induce retraction of LECs and thus, distortion of the integrity of the lymphatic walls (99). Gaps appearing in the structure of LVs facilitate the migration of tumor cells into LVs, and consequently promote metastasis towards draining LNs. In this context, a study conducted by Van de Velde et al. revealed that LECs exposed to human skin carcinoma cells secreted large amounts of pro-inflammatory IL-6 compared to control group (100). The tumor promoting effect was then neutralized with an anti-IL-6 antibody, indicating that IL-6 is an important factor contributing to tumor expansion (100).

Other cell type of special importance within the TME are tumor-associated macrophages (TAMs). Infiltration of immune cells within mammary tumors has been associated with poor prognosis (101). In agreement, a correlation between the presence of TAMs and the lymphatic proliferation in the peritumoral stroma was described (102). This phenomenon was elucidated by identification of TAM subsets secreting VEGF-C and VEGF-D and the elevated number of PDPN^+^ and LYVE-1^+^ microvessels present in the surrounding of the primary tumor, but not within the cancerous tissue. On the contrary, increased proliferation of CD34^+^ vessels were present both in the peritumoral stroma and within the tumor (102).

Intriguingly, a recent study conducted by Bieniasz et al. found that PDPN expression in TAMs was critical for their adhesion to LECs, which was necessary for their accumulation and activation in the proximity of LVs (103). This study revealed that PDPN binds to galectin 8 (GAL8), a secreted lectin produced by LECs, in a glycosylation-dependent manner, and this interaction promotes the activation of the pro-migratory integrin β-1, facilitating the attachment of TAMs to LECs (**Figure 3A**). Notably, PDPN-expressing macrophages (PoEMs) were found to promote lymphangiogenesis and local extracellular matrix (ECM) remodeling, enhancing LV growth and the subsequent lymphatic invasion by tumor cells (103) (**Figure 3A**).

An interesting study conducted in murine heterotopic and spontaneous tumor models demonstrated that LECs within the TME exhibit an increased expression of the major histocompatibility (MHC)-II, in association with augmented co-inhibitory signals (104) (**Figure 3A**). Moreover, tumor-associated lymphatics in human melanoma and breast cancer also upregulated MHC-II in comparison to normal LVs (104). Transgenic mice lacking LEC-specific MHC-II expression displayed attenuated heterotopic tumor growth, accompanied by elevated numbers of tumor-specific CD8^+^ and effector CD4^+^ T cells, as well as reduced numbers of T regulatory CD4^+^ cells in the TME (104). These results revealed that murine and human dermal LECs can uptake tumor antigens *in vitro*, and antigen-loaded LECs can induce antigen-specific CD8^+^ T cell proliferation, but not CD4^+^ T cell proliferation. Notably, the proliferative CD8^+^ T cells exhibited reduced effector function in the presence of antigen loaded LECs (104). Collectively, these findings suggest that LECs can serve as immunosuppressive cells in the TME through an MHC-II-dependent manner. However, further studies are necessary to determine whether this is due to direct tumor antigen presentation on MHC-II (104).

Accumulated evidence has shown that contribution of LECs to the establishment and maintenance of an immunosuppressive TME mainly relies on T cell tolerance induction, which is mediated by four major mechanisms (105): suppression of DC-mediated T cell activation; expression of MHC class I and II molecules; expression of PD-L1; and secretion of immunosuppressive cytokines (**Figure 3A**). Therefore, the influence of LECs on T cells may be indirect or direct. Indirect regulation of T cells is supported by a study conducted by Podgrabinska et al., which revealed that tumor necrosis factor (TNF)-α-stimulated LECs reduced CD86 expression in DCs through MAC-1/ICAM-1 interaction, which eventually suppressed DC-mediated T cell proliferation (106). On the other hand, direct modulation of T cell function is supported by the fact that LECs express MHC-I and II molecules, and therefore can induce CD8^+^ and CD4^+^ T cell tolerance, respectively (104).

T cells are activated within the tumor draining lymph nodes (TDLNs), where they acquire their effector functions to migrate toward the tumor (107). The migration of effector T cells from LNs to the tumor site plays a crucial role in determining the density and diversity of tumor-infiltrating T cells, which in turn affects the prognosis and efficacy of cancer immunotherapies (21, 108). In summary, this process requires T cells to first attach to the endothelium, followed by rolling, firm adhesion and activation on the endothelial surface, and finally extravasation through the blood vessel and LV wall into the tumor site (109). In a three-dimensional cell culture system without chemokine gradients, T cells move along the fibrillar collagen network through a process independent of integrin or protease activity. When activated CD8^+^ T cells are encapsulated in collagen hydrogels with distinct fiber alignment, CD8^+^ T cells move faster and more persistently in aligned collagen fibers than in non-aligned collagen fibers (110). Additionally, when naïve T cells are activated and become effector T cells (Teff) in LNs, Teff express high levels of CD44 and can bind to hyaluronic acid (HA). CD44 and its engagement with HA drives CD8^+^ T cells towards a terminal effector differentiation state that reduces their ability to form memory cells (111). The naïve CD4^+^ and CD8^+^ T cells or memory precursor effector cells are supported by fibroblastic reticular cells in the T cell zone through the production of IL-7, IL-15, and CCL19. Studies have shown that the activation of T cells through the T cell receptor (TCR) can be affected by mechanical force, as antigens linked to a stiff surface can impair TCR-mediated activation (112–114). Therefore, attracting immune cells to the TME is responsible for structural changes within the ECM (115), and the function of T cells can be regulated by their interactions with ECM structural components present in the TDLNs. In fact, tumor-associated ECM enables the formation of an immunosuppressive environment that favors immune evasion through various mechanisms, such as limiting immune cell migration, controlling polarization of myeloid cells or modulating T cell function (115, 116).

Tumor cells also interact with the ECM and cause changes that result in the alteration of its physical properties, increasing its density and stiffness (115). Pressure exerted by stiffened ECM on the surrounding tissues allows increased adhesion between cells and the ECM, as well as distortion of cell-cell contacts, ultimately leading to cell survival and growth (117, 118).

Regarding LECs present in TDLNs, it is worth highlighting that cLECs express the immunoregulatory CD73 and the lipid transporter CD36, both of which display pro-tumor properties (119). Studies in CD73 knock-out mice have demonstrated an increased expression of pro-inflammatory genes in LN LECs, leading to heightened recruitment of inflammatory DCs upon immunological challenge (120, 121). While no abnormal CD73 gene expression has yet been detected in chronic inflammatory diseases, this avenue of research holds immense promise. Moreover, afferent lymph often contains fatty acids, including low-density lipoproteins, which are transported via CD36 (69, 121).

Finally, the importance of miRNAs has been implicated in the crosstalk within the TME. A study by Zhou et al. has demonstrated that in case of cervical cancer, miR-1468-5p, a cancer-secreted exosome-encapsulated miRNA, promotes lymphatic PD-L1 upregulation and lymphangiogenesis, ultimately resulting in the impairment of lymphocyte T function. The mechanism by which exosomal miR-1468-5p exerts its effects involves the epigenetic activation of the JAK2/STAT3 pathway in LECs. This activation triggers an immunosuppressive environment that allows cancer cells to evade the anti-cancer immune response (122).

Overall, cancer cell, LEC and immune cell interactions, as well as their crosstalk with the tumor-associated ECM, contribute to the pro-tumor properties of the lymphatic vasculature and the suppression of the anti-tumor immunity.

### 4.2 Anti-tumor immune properties of LECs

A positive correlation between LV density and anti-tumor immunity has been described, thereby supporting a role of lymphatics in immune surveillance (123). Promoting the recruitment and activation of anti-inflammatory immune cells in the TME plays also a crucial role in the induction of anti-tumor responses. The secretion of adhesion molecules is responsible for determining the migration of those cells conditioning the anti-tumor response towards the primary tumor site (**Figure 3B**). LECs also secrete anti-inflammatory cytokines and chemokines, which modulate the TME and create unfavorable conditions for tumor growth and progression (76) (**Figure 3B**).

On the other hand, LECs from the subcapsular sinus (LECs II) have been reported to express chemokines, co-stimulatory and co-inhibitory molecules that play crucial roles in T cell function (69). Among these molecules, CCL20 (also known as MIP-3A) has been shown to be highly expressed in LECs II and is the only known ligand for the chemokine receptor CCR6 (124). CCR6 expression has been identified on a variety of immune cells, including Th17 cells, which are memory T helper cells that produce IL-17A (124, 125). Th17 cells are supposed as a double-edged sword in cancer, since they can increase tumor progression by activating angiogenesis and immunosuppressive activities, but also, they can modulate anti-tumor immune responses through recruiting immune cells into tumors, stimulating effector CD8^+^ T cells, or by altering toward Th1 phenotype and producing interferon (IFN)-γ (126). Additionally, CCR6 is also expressed on gamma delta (γδ) T cells, which have been implicated in the pathogenesis of several chronic inflammatory diseases (125), and in IL-17A^+^ innate-like lymphocytes in the mouse LN subcapsular sinus, involved in innate responses against lymph-borne pathogens (127). Furthermore, LECs II express chemokines such as CXCL5 and CXCL1, which are known to attract neutrophils (71).

LECs have been shown to promote the differentiation and activation of DCs, which are important antigen-presenting cells that bridge innate and adaptive immunity (128). The anti-tumor role of LECs includes the induction of DC migration towards TDLNs and consequent DC-mediated antigen presentation to naïve T cells (129). In tissues with inflammation, where DCs migrate extensively via lymph, the transmigration process relies on adhesion mechanisms that are mediated by integrins (130). A study in human melanoma has described that LECs support the recruitment of naïve T cells in the local TME through secretion of CCL21, which is induced in response to VEGF-C (131).

Recent research has demonstrated that primary cultured LECs, when exposed to inflammatory cytokines and contact sensitizing agents, rapidly upregulate the key integrin counter-receptors ICAM-1 and VCAM-1, together with a host of other adhesion molecules, that facilitate leucocyte endothelial transit (132). These adhesion molecules include E-selectin and a variety of different chemokines that serve as attractants for DCs, monocytes, lymphocytes, and neutrophils (133, 134). Additionally, studies using ICAM-1 and VCAM-1 blocking monoclonal antibodies have shown that these molecules are critical for adhesion and transmigration of bone marrow DCs across inflamed LEC monolayers *in vitro* (132). Furthermore, the use of these blocking antibodies has demonstrated that they impede the entry and trafficking of endogenous DCs to TDLNs in murine models of skin hypersensitivity and dermal vaccine-induced T cell immunity (134, 135).

In conclusion, LECs play a crucial role in the TME by actively participating in the regulation of immune responses against tumors. Nevertheless, the immunosuppressive signals from LECs can also inhibit immune cell activation, impair effector functions, and promote immune tolerance towards the tumor. The dual nature of the role played by LECs highlights the complexity of the immune responses within the TME and underscores the need for a comprehensive understanding of LEC-mediated immunomodulation in the context of tumor immunity.

## 5 Cancer -lymphatic- immune cell crosstalk during metastatic dissemination

### 5.1 Initial metastatic invasion in primary tumors

Metastasis is a complex process by which cancer cells from a primary tumor disseminate and colonize distant organs (136). Despite being the cause of most of the deaths related to cancer (137), there is still a poor understanding of the mechanisms involved in this phenomenon. The lymphatic system plays an important role in several steps of cancer metastasis, since tumor cells can invade LVs and be transported to TDLNs, where they can survive, grow and even spread to distant organs (138). The relevance of the lymphatic system in the process of metastasis is shown by the correlation that exists between LV density, metastasis and poor prognosis (139). Moreover, in most of tumor types, LN metastasis is a negative prognostic marker (14).

**Figure 4:**
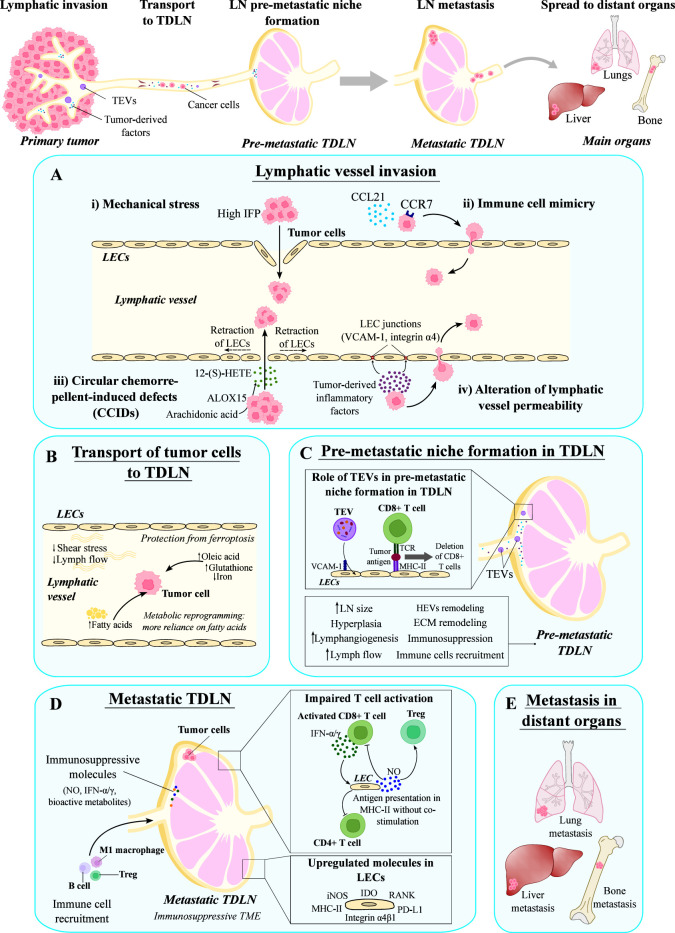
Role of the lymphatic system in the metastatic dissemination. The lymphatic system is involved in several steps of the metastatic process, such as the initial invasion of tumor cells into lymphatics, their transport to tumor draining lymph nodes (TDLNs), and the formation of distant metastasis in the main organs. **(A)** Tumor cells can invade lymphatic vessels by several mechanisms: (i) generation of mechanical stress due to the high interstitial fluid pressure (IFP) that disrupts the lymphatic vessel wall; (ii) mechanisms involved in immune cell migration, such as the CCL21/CCR7 axis; (iii) formation of circular chemorepellent-induced defects by generating 12-(S)-HETE; and (iv) alteration of the lymphatic vessel permeability by producing factors that destabilize lymphatic junctions. **(B)** Tumor cells are transported to TDLNs by lymphatic vessels, where lymph properties promote their survival and metabolic reprogramming. **(C)** Before the arrival of tumor cells, TDLNs suffer changes induced by tumor-derived factors transported in extracellular vesicles (TEVs) that prepare the TDLN and form the pre-metastatic niche. **(D)** Metastatic TDLNs are characterized by an immunosuppressive microenvironment due to the impaired T cell activation and the presence of immunosuppressive molecules. In this context, LECs upregulate several molecules that contribute to immunosuppression, such as IDO, RANK, PD-L1, Integrin α4β1, MHC-II and iNOS. **(E)** Cancer cells in metastatic TDLNs can spread to distant organs giving rise to metastasis. LECs, lymphatic endothelial cells; LN, lymph node.

Invasion of LVs by cancer cells may be accomplished through different mechanisms (139) (**Figure 4**). In a first mechanism, cancer cells can access LVs in a passive manner by generating mechanical stress that disrupts the LEC wall (139). Then, cancer cell invasion into LVs is facilitated by the high interstitial fluid pressure inside the tumor (138) (**Figure 4A**). In an additional process, the immune system is also involved, as cancer cells express chemokine receptors and can use the chemokine gradients employed in leukocyte homing to access LVs, which is named immune cell mimicry (139) (**Figure 4A**). It has been previously described that CCL21/CCR7 axis plays an important role in directing CCR7-expressing cancer cells to LVs (140). This pair promotes growth and metastasis of many tumor types, including esophageal, melanoma, breast, thyroid, colorectal, head and neck cancers (141–147). Other chemokine ligands important during the metastatic process include the CXCR3 ligand CXCL10 (in melanoma and colorectal cancer) (148, 149), the CXCR4 ligand CXCL12 (in multiple types of cancer) (150, 151) and CCL1 (in melanoma) (152). The interplay between CCL5 and CCR5 present on breast cancer cells enables the formation of metastatic niches, and thus facilitates the metastatic cascade (153). On the other hand, the chemokine receptors present on the LEC surface are mainly involved in the processes of tumor-specific lymphangiogenesis and lymphatic metastasis, e.g. CXCR4 targeting CXCL12 present on both tumoral and stromal cells, CXCR2 with an affinity for CXCL5 in melanoma cells, and CXCL1 expressed by LECs in gastric cancer (87, 154, 155).

Another mechanism of cancer cell invasion is the generation of circular chemorepellent-induced defects (CCIDs) in the wall of the LVs (14). These lesions are formed as a result of the release of the chemorepulsive compound 12-hydroxyeicosatetraenoic acid (12-(S)-HETE) by arachidonate 15-lipoxygenase (ALOX15)-expressing cancer cells. ALOX15 is an enzyme that transforms arachidonic acid into 12-(S)-HETE, which induces a signaling cascade that results in the retraction of LECs, the formation of CCIDs in the lymphatic walls, and the entry of cancer cells into LVs (99, 156) (**Figure 4A**). Additionally, cancer cells can also alter LV permeability by destabilizing lymphatic junctions (139). In this context, upregulation of VCAM-1 and integrin α4 expression in tumor-associated LECs, due to the presence of tumor-derived inflammatory factors, leads to weakened LEC junctions, further contributing to increased lymphatic permeability and cancer cell penetration into LVs (157) (**Figure 4A**).

TAMs have been reported to play important roles during lymphatic-associated metastatic progression, as they induce lymphangiogenesis through VEGF-C/D secretion following stimulation by tumor cell-derived IL-1α and IL-8 (93). In the case of cervical squamous carcinoma, the impact of the lymphatic remodeling in the hypoxic TME was investigated (158). A unique pattern termed LVs encapsulated by TAMs (LVEM), where TAMs surround LVs, contribute to enhanced metastatic capacity and early LN metastasis (158). Mechanistically, hypoxic TAMs release IL-10 promoting lymphangiogenesis and LVEM formation by upregulating *Sp1* in LECs. This process establishes a positive feedback loop involving CCL1, thus facilitating the recruitment of TAMs and tumor cells, and reinforcing LVEM formation (158).

Interestingly, the lymphatic marker LYVE1 has been found to be involved in immune and cancer cell migration (159, 160). Recent studies have shown that LYVE1-positive tumor cells acquire a LEC-like phenotype and utilize this receptor for lymphatic spread (161). Targeting LYVE1 has demonstrated therapeutic potential by impairing cancer-related vasculature growth and reducing metastasis (161).

Once inside LVs, properties of the lymph and lymph flow favor the maintenance of cancer cells while they are transported to TDLNs (14) (**Figure 4B**). Contraction of tumor-draining LVs is impaired because of the presence of cancer-associated myeloid-derived suppressor cells (MDSCs), which alter nitric oxide (NO) dynamics needed for LV contraction (162). Consequently, the slower flow and reduced shear stress allows cancer cell survival (138) (**Figure 4B**). Moreover, lymph contains high amounts of oleic acid and glutathione, and low levels of iron, which has been shown to protect cancer cells (i.e. melanoma cells) from ferroptosis (163). In addition, the presence of fatty acids in the lymph induces a metabolic reprogramming in cancer cells (164). Altogether, the conditions of physical and chemical properties of the lymph favor the survival of tumor cells.

### 5.2 Metastatic dissemination to lymph nodes

Around 25% of metastatic tumors observed in distant locations can be traced back to the dissemination of cancer cells through LN metastasis (165). Increasingly, new findings indicate that the process of tumor metastasis could potentially be initiated during early stages of tumorigenesis (166).

Interestingly, a correlation has been discovered between the augmentation of angiogenesis and lymphangiogenesis in the sentinel lymph node (SLN), the first TDLN in which cancer cells from the primary tumor appear, and the occurrence of distant metastasis, as well as the survival outcomes of melanoma patients (167). In fact, in a comprehensive study involving 8.562 patients, it was established that the status of the SLN is a vital prognostic determinant among individuals diagnosed with stage IIB/C melanoma (168).

The immune microenvironment within the LN is governed by a diverse array of immune cells, including macrophages, DCs, T cells, B cells, and non-immune cells like fibroblastic reticular cells, BECs, and LECs (1). Before arrival of cancer cells, TDLNs suffer changes such as increased size, hyperplasia or recruitment of immune cells (14). A range of biological features has been described, especially in the SLNs. These modifications encompass the stimulation of lymphangiogenesis, an increase in lymph flow (169), structural remodeling of high endothelial venules (HEVs) (170, 171), enhanced recruitment of myeloid cells, and a reduction in effector lymphocytes (172) (**Figure 4C**). These combined elements contribute to the establishment of the pre-metastatic niche, term used to refer to the microenvironment that facilitates the further entry and survival of cancer cells (16). In both mouse solid tumor models and human cancers, the growth of lymphatic sinuses within TDLNs has been observed and it is associated with altered lymph flow, having crucial implications for tumor growth, and metastasis to LNs and distant organs (173). The expansion of lymphatic sinuses in TDLNs may hinder anti-tumor immune responses, while increased lymph drainage could facilitate metastasis (173). Additionally, remodeling of the ECM in TDLNs is also thought to occur as part of the pre-metastatic niche formation, but the mechanisms that underlie this process and their molecular players remain to be uncovered (16).

A single-cell analysis carried out in cells isolated from murine breast cancer models showed changes in immune modulation and metabolism in TDLNs (174). Moreover, a study performed in cervical neoplasms revealed that pre-metastatic SLNs exhibited a notable increase in LV density and specific distribution patterns compared to non-SLNs (175). This study also revealed a heightened inflammatory profile in pre-metastatic SLNs, characterized by increased expression of CD8^+^, Foxp3, CD20, and PD-1. These findings suggest that both lymphatic and immune responses contribute to the development of a unique pre-metastatic microenvironment in TDLNs (175). Furthermore, the densities of CD20^+^ B cells and PD-1 expressing germinal centers in TDLNs were positively correlated with LV density, indicating the involvement of humoral immune responses in the pre-metastatic process (175).

The TDLN modifications mentioned above, induced by tumor-secreted factors, prepare the LN for the arrival of cancer cells, creating the pre-metastatic niche (16). Although signals involved in this TDLN preparation remain to be completely understood, tumor-derived extracellular vesicles (TEVs) can serve as carriers of multiple tumor-derived molecules (nucleic acids, proteins and metabolites) that play a key role in the remodeling of TDLNs (176, 177). TEVs can be transported by LVs to TDLNs, where they may be retained. LN LECs can take up TEVs through a mechanism that involves VCAM-1, resulting in the expansion of lymphatics and changes in the transcriptome of LECs (178). Moreover, TEVs may contain tumor antigens that can be presented to CD8^+^ T cells by LECs leading to their deletion, and thus contributing to the inhibition of tumor immunity (178) (**Figure 4C**).

Other molecules have been found to be upregulated in tumor-associated LECs in metastatic TDLNs. These molecules include the inducible nitric oxide synthase (iNOS) and indoleamine 2,4-dioxygenase (IDO). iNOS catalyzes the synthesis of NO, a known inflammatory mediator with immunosuppressive properties. *In vitro* production of NO by LECs requires direct interaction with activated T cells and consequent T-cell mediated secretion of IFN-γ and TNF-α (179). Once produced, NO suppresses T cell and induces Treg proliferation (180) (**Figure 4D**). A role of NO in immune metabolism has been already described, since it induces glycolysis and inhibits cytochrome c oxidase complex IV on T cells, thereby presumably controlling mitochondrial respiration (181). On the other hand, IFN-γ and TNF-α can also stimulate the expression of IDO (**Figure 4D**). IDO is the first and rate-limiting enzyme of tryptophan catabolism, and its activity can increase the levels of bioactive metabolites involved in Treg differentiation and T cell anergy (180). This immunosuppressive effect is also evoked by IDO-mediated tryptophan insufficiency, which results in metabolic stress (182).

Despite LNs having a central role in the immune response, metastatic TDLNs present an environment of immunosuppression that contributes to metastasis (174). Importantly, a study recently conducted by Reticker-Flynn et al. has provided compelling evidence that the invasion of cancer cells into LNs induces prolonged interferon signaling, consequently initiating antigen-specific immune tolerance to facilitate the progression of distant metastasis (183). Both stromal and immune cells in TDLNs suffer changes at the transcriptomic level that are involved in the adaptation of the TDLNs to favor LN metastasis (174). Interestingly, metastatic TDLNs microenvironment is different from that of the primary tumor or other metastatic organs (184–186). In agreement, multiple mechanisms for immunosuppression in metastatic TDLNs have been proposed, such as modulation of T cell activation and function or NK cell activity (184, 187). Among the immunological reprogramming observed in metastatic TDLNs, it is worth mentioning that several immune cell types seem to increase in TDLNs, such as M1 macrophages in oral squamous cell carcinoma (OSCC) (185), and B cells and Tregs in breast cancer (184, 188). Interestingly, a metastasis-promoting role of B cells in pre-metastatic TDLNs has been described (188). In fact, B cells are increased in TDLNs and they produce pathogenic IgG that interacts with heat shock protein family A member 4 (HSPA4), activating the NF-κB pathway that mediates the activation of the CXCR4/SDF1α axis (188). On the other hand, Tregs in TDLNs have been shown to contribute to the immunosuppressed microenvironment by modulating NK cells (184).

As mentioned in the previous sections, DCs are highly specialized antigen-presenting cells that act as key regulators of the host immune system’s ability to target and eliminate cancer cells (189). They play a crucial role in initiating cellular immunity, which is essential for initiating a robust immune response against cancer (166). Previous investigations have compellingly demonstrated that the effectiveness of T cells in combating cancer is markedly reduced in the absence of DCs (190). In the context of the SLN, the activation of T cells by DCs is significantly impaired due to the influence of cancer cells, which can disrupt this process through direct cell-to-cell contact or by releasing factors such as TGF-β (191) and VEGF (192). At present, VEGF-A and VEGF-C have emerged as the extensively studied soluble factors associated with the development of a pre-metastatic microenvironment within the SLN (193, 194). The modulation of LECs through the VEGF-C-PI3K pathway plays a vital role in tumor-associated lymphangiogenesis. This signaling pathway facilitates the upregulation of integrin α4β1 on LECs, consequently attracting VCAM-1-expressing tumor cells (195) (**Figure 4D**).

LECs found within the SLN exhibit heightened expression levels of the receptor activator of nuclear factor kappa-B (RANK). Concurrently, stromal reticular cells activate LECs via RANK ligand (RANKL), instigating a notable remodeling process (196) (**Figure 4D**). Furthermore, this reprogramming of LECs exerts an impact on their immunomodulatory capabilities. In a physiological context, LECs possess the ability to present an array of peripheral tissue antigens through MHC-I molecules (86). This distinctive presentation triggers immune tolerance and effectively modulates the proliferation of CD8^+^ T cells by engaging PD-1/PD-L1 signaling (187). Moreover, CD8^+^ T cells increase the expression of PD-1 in the presence of IL-8 in gastric cancer, which contributes to immunosuppression in TDLNs (197). LECs also interact with CD4^+^ T cells (198). *In vitro* studies using LECs isolated from LNs of cancer patients show that activated LECs upregulate MHC-II molecules (**Figure 4D**) but do not express co-stimulatory molecules such as CD80 and CD86, failing to activate CD4^+^ T cells (199). Moreover, LECs upregulate the expression of inhibitory signals such as PD-L1 and IDO when stimulated with IFN-γ (199) (**Figure 4D**). CD4^+^ T cells co-cultured with activated LECs reduced their production of activation cytokines (IL-2, IL-10, IFN-g), confirming the role of LECs in the suppression of CD4^+^ T cells in TDLNs (199).

Upon arrival to TDLNs, cancer cells can disseminate to distant organs giving rise to metastasis (**Figure 4E**). Despite the importance of lymphatics in metastasis and cancer progression, the exact mechanisms responsible for cancer cell dissemination from primary tumors to TDLNs, and further to peripheral organs through tumor-associated LVs, are incompletely understood and remain to be fully elucidated.

## 6 Lymphatics and immunity in the context of cancer immunotherapy

An active field of research within lymphatic vascular biology arose from the concept that LVs might not only promote tumor progression via enhanced metastasis but also through contribution to the establishment and maintenance of an immunosuppressive TME (4, 129). To date, it is also accepted that lymphatics may play a dual role in the TME, as both anti-tumor and pro-tumor immune responses have been ascribed to tumor-associated LECs, suggesting the promising role of tumor LECs in potentiating immunotherapy.

The immunosuppressive phenotype of tumor-associated LECs is also defined by the expression of PD-L1, a checkpoint inhibitor that interacts with its receptor PD-1 on T cells to induce peripheral T cell tolerance. Signaling through PD-L1 impedes IL-2R upregulation on CD8^+^ T cells, and consequent loss of IL-2 signaling, which is necessary for CD8^+^ T cell survival and results in apoptotic death (200). Considering the emergence of checkpoint inhibitors as powerful cancer immunotherapies, these findings have revealed a promising possibility to expand lymphangiogenic-based therapies towards PD-L1/PD-1 targeting. Indeed, the benefits of targeting immune checkpoint inhibitors to TDLNs have already been confirmed (201, 202). Reports on glioblastoma, one of the most aggressive types of cancer, describe a positive correlation between tumor clearance and VEGF-C-mediated meningeal lymphangiogenesis (203). In fact, the existence of the central nervous system immune privilege impedes checkpoint inhibitors to reach the brain and access the tumor. In line with this, a recent study has revealed that VEGF-C improves the anti-tumor effect of anti-PD-1/CTLA-4 antibodies (203). Thus, since VEGF-C-induced lymphangiogenesis has been described to improve the response to PD-1/CTLA-4 therapies in glioblastoma, LECs are now regarded as important mediators in potentiating immunotherapy (21, 203).

The interplay between LECs and T cells harbors a promising opportunity for the treatment of cancer. In fact, recent findings have provided insight into how cytotoxic (CD8^+^) T cells interact with LECs in the TME, showing a dose-dependent effect of T cells on the immune response. Garnier et al. showed that, while IFN-γ released by T cells initially induced LV immunosuppression, elevated T cell densities eventually dampened this effect and induced LEC apoptosis (204). At higher T cell densities, IFN-γ stimulated the cross-presentation of tumor antigens by LECs following T cell-mediated tumor death, which subsequently lead to LEC apoptosis and reduction of tumor-associated LV density and metastasis (204). Although the mechanism underlying these findings remains to be further studied, these results offer an optimistic possibility to enhance anti-tumor T cell responses as a strategy to combat cancer.

While T cell-mediated immune response within TDLNs has received more attention, the role of B cells remains poorly understood. Nevertheless, a recent study conducted by Louie and colleagues has established a connection between tumor antigen-specific B cell maturation and tumor growth (205). Using B16F10 melanoma cells and a mouse model of breast cancer, the authors reported that tumors reconstructed TDLNs by disruption of the subcapsular sinus macrophage layer, which is a route for antigens to enter the germinal centers (GCs). Consequent entry of tumor-associated antigens into the GCs promoted B cell activation and maturation, which positively correlated with tumor growth and metastasis (205). While the role of B cells in cancer immunity remains controversial, this study highlights the connection between tumor progression in TDLNs and B cell activity, shedding light on future studies that may contribute to a deeper understanding of immunity within TDLNs and to the development of cancer immunotherapies.

The connection between immunity and lymphatics is receiving increasing attention, and nowadays, it constitutes one of the most active areas of research within tumor-associated lymphatics. The therapeutic opportunities arising from immune checkpoint blockade and the recently described PD-L1-mediated immunomodulatory function of LECs holds promises in cancer immunotherapy. Nevertheless, further efforts are needed to study the crosstalk between cancer cell, lymphatics and immune cells in order to identify new targets for cancer immunomodulation. Future immunotherapies will therefore benefit from the study of the interplay between LECs and the immune system in pathological conditions, and from understanding the molecular and cellular bases underlaying the reprogramming that these cell types may undergo to provide an immunosuppressive niche in primary tumors and TDLNs.

## 7 Concluding remarks and future perspectives

The immunomodulatory functions of LECs in the TME are complex and multi-faceted. LECs have recently emerged as key players in the regulation of the TME, with scientific evidence indicating that they can modulate the immune response and promote the recruitment and activation of immune cells. They play a crucial role in the regulation of LV formation, immune cell trafficking and immune cell activation; however, they can also exert a suppressive effect on the immune response. Further research is necessary to understand the exact function of LECs and their interactions with other elements of the TME, especially in the context of the immune response regulation.

The rationale underlying the paradoxical role played by LECs in cancer immunomodulation, by facilitating anti-tumor immunity and, at the same time, inducing immune tolerance, remains unknown. Nevertheless, it might be partially explained by the fact that even though tumor-associated LVs initially contribute to immune cell recruitment and adaptive immune response initiation, the immunosuppressive role of LECs in the TME eventually dampens preexisting anti-tumor immunity. Therefore, it has been proposed that LVs may play a dichotomic role on tumor immunity and it might be temporally regulated (129). Besides, the role of LECs in tumor immunomodulation might be context dependent. In any case, it is likely that the global balance between LEC-mediated pro-tumor and anti-tumor immune responses finally determines the fate of tumor progression.

While the extensive research in oncology and immunology leads to the development of immunotherapies, there are still many aspects that need to be improved. Currently available therapies targeting lymphatic system include utilization of cytokines (e.g., IL-2 or IFN-α), cell-based therapies or immune checkpoint blockade. Unfortunately, these therapies are known to cause adverse effects and to be ineffective, as in many patients the results are not permanent (206, 207). Aside from immunotherapy, other approaches in cancer management rely on the importance of the lymphatic system. Techniques such as SLN biopsy and lymphatic mapping (e.g., with fluorescent dyes and radio-labelled tracers) are becoming increasingly important tools in the surgical management of cancer as they allow for the identification of the specific LVs that drain a cancerous tissue. This helps determine the extent of lymphatic spread, and hence, to make informed decisions about surgical procedures. These techniques constitute promising bases for establishing future guidelines for physicians and other healthcare providers.

Advances in understanding LEC behavior and interactions within the TME, cancer initiation, tumor progression and metastatic spreading, might constitute a future basis underlying the development of novel therapies aiming not only at cancer treatment, but potentially also prevention of metastasis. Besides cancer therapy, setting LECs as important druggable components could potentially improve drug delivery systems in localized and systemic inflammation, as well as enhance lymphatic drainage and reduce edema. However, the heterogeneity displayed by distinct LEC subsets, and the dynamic nature of intercellular interactions raise questions regarding current state of knowledge about the exact function of these cells. Additionally, LEC behavior might possibly undergo changes in various stages of the disease and be affected as the patient undergoes chemotherapy and appears resistance to treatments. Nevertheless, given the wide range of functions that LECs can potentially perform, they undoubtedly should be considered important stromal component of the TME. Elucidation of factors conditioning the nature of their immunomodulatory properties could be the key to harnessing their potential to improve anti-tumor immunity and search for new treatments in other human diseases in which lymphatics are involved.

## Author contributions

CV-P, EK, and MG-C wrote the initial version of the manuscript. CV-P, EK, and MG-C created the figures. MG-C revised the final version of the manuscript. All authors contributed to the article and approved the submitted version.
